# Video data for the cognitive mapping process of NeuroBayesSLAM system

**DOI:** 10.1016/j.dib.2020.105637

**Published:** 2020-04-30

**Authors:** Taiping Zeng, Bailu Si

**Affiliations:** a Institute of Science and Technology for Brain-Inspired Intelligence, Fudan University, Shanghai, China; b Key Laboratory of Computational Neuroscience and Brain-Inspired Intelligence (Fudan University), Ministry of Education, China; c School of Systems Science, Beijing Normal University, 100875, China

**Keywords:** Cognitive mapping, Multisensory integration, Bayes, Grid cells, Head-direction cells

## Abstract

Simultaneous localization and mapping (SLAM), which addresses the problem of constructing a spatial map of an unknown environment while simultaneously determining the mobile robot's position relative to this map, is regarded as one of the key technologies in mobile robot navigation. This data article presents four raw video files, demonstrating the mapping and localization processes of NeuroBayesSLAM, a neurobiologically inspired SLAM system, on two publicly available datasets, namely the St Lucia suburb dataset and the iRat Australia dataset. The cognitive mapping process was recorded by a free screen recorder software on ubuntu Linux system. Neural activities of the head-direction cells and the grid cells, the local view templates of visual scenes, and experience map were included. These data envision the possibility of transferring the multisensory integration mechanism found in the spatial memory circuits of the mammalian brain to develop intelligent cognitive mapping systems for indoor and large outdoor environments as in the research article “NeuroBayesSLAM: Neurobiologically Inspired Bayesian Integration of Multisensory Information for Robot Navigation” Zeng et al., 2020.

**Specifications Table**

**Table:** 

**Subject**	Neuroscience
**Specific subject area**	Computational Neuroscience
**Type of data**	Video
**How data were acquired**	Acquired with a screen recorder software (SimpleScreenRecorder)
**Data format**	Raw
**Parameters for data collection**	Video data were recorded at 30 frames per second by a free screen recorder software. The video frames are further downsampled to produce video files of no more than one minute in length.
**Description of data collection**	When the NeuroBayesSLAM system is running on publicly available datasets based on Robot Operating System (ROS), the screen on Ubuntu is recorded as the video data.
**Data source location**	Institution: Fudan University City/Town/Region: Shanghai Country: China Latitude and longitude (and GPS coordinates) for collected samples/data: 31.29889° N, 121.49917° E
**Data accessibility**	With the article
**Related research article**	Zeng et al. “NeuroBayesSLAM: Neurobiologically Inspired Bayesian Integration of Multisensory Information for Robot Navigation”, Neural Networks, 126: 21-35, 2020

**Value of the Data**

• The videos presented in this brief demonstrate the performance of a cognitive mapping system, namely NeuroBayesSLAM, on two publicly available datasets.
• The conflict between vestibular cue and visual cue is resolved by the competitive dynamics and also explicitly presented in the videos.
• The videos provide comparison and benchmark against other brain-inspired SLAM methods.
• The dynamic process of neural activities, local view scenes, and experience map over time can help better understand the neural computational mechanisms of head-direction cells and grid cells and their implications for developing intelligent navigation system.

## 1 Data Description

The dataset comprises four raw video files recorded using a free screen recorder software (SimpleScreenRecorder) on ubuntu system when the NeuroBayesSLAM system is demonstrated on two publicly available datasets, namely the St Lucia suburb dataset and the iRat Australia dataset [1]. The videos are encoded in MP4 format. Each video frame is composed of sub-panels visualizing the neural activities of the head-direction cells and the grid cells, input visual scenes, the local view templates of visual scenes, the best-matched template, and the experience map.

Video S1 shows the mapping process for the St Lucia suburb dataset. Video S2 shows the plastic remapping process when a loop is successfully closed with strong reliability of visual cues. While, weak reliability in visual landmarks fails to complete the loop closure, as shown in Video S3. Video S4 shows the mapping process for the iRat Australia dataset. Supplementary videos are with the article.

## 2 Experimental Design, Materials, and Methods

The demonstration presented in the video data is based on the NeuroBayesSLAM system [2]. To record the running process, a computer with two monitors (A, B) is set up. Every ROS node is separately running in a different terminal in the Ubuntu Linux system on the monitor A. The visualization windows of the NeuroBayesSLAM system are put on monitor B arranged by mouse drag, including neural activities of the head-direction cells and the grid cells, the local view templates of visual scenes, and the experience map. After the simple screen recorder software is set up on monitor A and is started to record the screen of monitor B, the rosbag node of the NeuroBayesSLAM system starts to read the datasets and sends ROS messages. The screen recorder software saves the complete mapping processes for the St Lucia suburb dataset into Video S1 and for the iRat Australia dataset into Video S4 respectively.

In order to demonstrate the competitive dynamics between the integrator cells and the calibration cells of the NeuroBayesSLAM system during the cue conflict resolution process, a loop in the St Lucia suburb dataset is selected to record in finer time resolution. We varied the strength of the injection current from visual cue to show the role of visual cues in plastic remapping process. The injection parameters with smaller energy are for weak/unreliable visual cues. The injection parameters with bigger energy are for strong/reliable visual cues. With strong visual cue, the integration of the visual landmark information generates a loop closure successfully (Video S2). In contrast, weaker visual landmarks fail to create a loop closure (Video S3).

## Declaration of Competing Interest

The authors declare that they have no known competing financial interests or personal relationships which have, or could be perceived to have, influenced the work reported in this article.

## Appendix Supplementary materials

Image, video 1

Image, video 2

Image, video 3

Image, video 4
